# The prevalence of infertility in 20-49 years women in Yazd, 2014-2015: A cross-sectional study

**Published:** 2018-11

**Authors:** Masoud Mirzaei, Nasim Namiranian, Razieh Dehghani Firouzabadi, Somaye Gholami

**Affiliations:** 1 *Yazd Cardiovascular Research Center, Shahid Sadoughi University of Medical Sciences, Yazd, Iran.*; 2 *Diabetes Research Center, Shahid Sadoughi University of Medical Sciences, Yazd, Iran.*; 3 *Research and Clinical Center for Infertility, Yazd Reproductive Sciences Institute, Shahid Sadoughi University of Medical Sciences, Yazd, Iran.*

**Keywords:** Infertility, Reproductive sterility, Yazd, Iran

## Abstract

**Background:**

Infertility is a serious health problem that affects the individual, her/his family, and the community. Infertility is defined as failure to achieve clinical pregnancy after at least 12 months of unprotected coitus.

**Objective:**

The purpose of this study was to investigate the prevalence of primary and secondary infertility and the associated factors in Yazd Greater Area during 2014-2015.

**Materials and Methods:**

This is a cross-sectional analytic study using Yazd Health Study data which was conducted on 10,000 people. We studied 2611 women between 20-49 yr old who lived in Yazd Greater Area. Data were collected using a validated questionnaire. Anthropometrics were collected using standardized instruments.

**Results:**

Among women participating in the study, 135 cases of infertility were documented and the overall prevalence of infertility was 4.73% (95% CI: 3.94%-5.59%), among them 2.6% (95% CI: 2.4-3.8%) had primary and 2.1% (95% CI: 1.8-3.4%) had secondary infertility. In this study, infertility was significantly correlated with age (r=0.051, p=0.032), educational level (r=-0.41, p=0.001), body mass index (r=-0.012, p=0.018), waist circumference (r=0.027, p=0.022), history of abortion (r=0.099, p=0.026), and family history of infertility (r=0.121, p=0.001).

**Conclusion:**

The results of our study showed that the prevalence of infertility among women living in Yazd was lower compared to the other regions in Iran. Female factors were the main cause of infertility in central part of Iran.

## Introduction

Infertility is defined as no pregnancy after 1 yr unprotected coitus (1). Potential infertility is considered as a serious health problem worldwide. Infertility affects the individual, family and community through physical, psychological, social and economic consequences. Social consequences include couples' argument, violence, stigma, isolation and divorce (2). Over the last decade, the annual increase in assisted reproductive technologies, has been approximately 5%-10% in countries, which in addition to the economic burden, their effectiveness is of high importance. According to a recent studies report "assisted reproductive technology procedures have evolved to incorporate complex ovarian stimulation protocols, in vitro treatment of gametes including intracytoplasmic sperm injection, extended embryo culture, cryopreservation of embryos, and more recently in vitro maturation of oocytes" (3-5). 

The causes of growing infertility may include; changing family circumstances, having a child in later age, the excessive use of contraception, illegal and legal abortion, adverse social conditions, climate related factors, geographical areas and possibly genetic variation (6). Infertility has negative consequences on demographic, socio-economic and health. Epidemiological studies on infertility help health policy makers to implement effective infertility prevention and treatment policies. There are limited studies about descriptive and analytical epidemiology of infertility in Iran particularly at the individual and population levels (7). 

Epidemiological studies estimated the infertility prevalence in reproductive age between 5-30% worldwide (8-10). This wide range of prevalence was reported because of different geographical areas with different definitions of infertility, the variety of studied samples and the method of prevalence calculation (11). In Iran, various epidemiological studies carried out. A meta-analysis study in Iran was conducted on 13 Iranian studies during 2003-2011 which reported the overall infertility prevalence 13.2% (8-18.3%). However, some of the included studies qualities were questionable. The only large study to investigate the prevalence of infertility in Yazd province was conducted on 2004-2005. That study reported the prevalence of infertility among 5200 couples living in rural and urban areas of Yazd province 5.52% (12). 

The present study intends to investigate the prevalence of infertility and its predictive factors in women 20-49 yr old who lived in Yazd 10 yr after that study.

## Materials and methods

The present study is a cross-sectional analytic study using Yazd Health Study (YaHS) data in 2014-2015 (13), conducted on 10,000 residents aged between 20-69 yr old. The sampling method was population-based, random and multi-stage stratified. 

Data were collected using a validated questionnaire. Anthropometrics were measured by trained researchers using standardized instruments. The anthropometric indices including weight, height, and waist circumferences were measured according to standard criteria and calibrated measurement tools. Body mass index (BMI) was defined based on weight (kg) ratio on height squared (m^2^) and its limits were determined according to the standard criteria. All of data gathering were based on YaHS study protocol. Details of the study were published elsewhere (13). Questions about infertility and its related factors were asked from women who participated in the study. Infertility is defined as no pregnancy after 1 yr unprotected coitus, primary infertility is defined as failure to achieve clinical pregnancy after 12 months or more unprotected coitus (1). The secondary infertility was defined as inability to be pregnant after first pregnancy (14). Out of 9965 participants, 4989 were women and 2611 were in the reproductive age (i.e.20-49 yr). In YaHS study protocol, age is considered as the categorical variable; 20-29, 30-39, 40-49, yr old. So the studied samples between 20-49 yr were included in our study.

**Ethical consideration**

This study was approved by the Ethics Committee of School of Public Health, Shahid Sadoughi University of Medical Sciences, Yazd, Iran (IR.SSU.SPH.REC. 1396.135 on 20.12.2017) and informed consent was obtained from all participants.

**Statistical analysis**

Descriptive statistics were reported as frequency and percentage. The Pearson correlation with r and Chi-square analysis with a significant level of 0.05 were used. Also, the infertility prevalence which is reported in this study is age adjusted according to the Yazd population age distribution in 2011 national census. All of the calculations were statistically analyzed using the Statistical Package for the Social Sciences (SPSS) version 20.

## Results

Among 9965 participants in the YaHS study, 2611 women aged 20-49 yr were included. The baseline characteristic of participating women in the study is presented in table I. The age-adjusted prevalence of infertility (95% confidence interval) in the 20-49 yr population was 4.73% (CI=3.94-5.59%) (Table II). Infertility rates in women aged 20-29 yr was 3.7 (3.11%-4.61%), in 30-39 yr old was 5.3% (4.54-6.31%), and in 40-49 yr old was 6.1% (5.25%-7.14%), and this difference was statistically significant (p=0.032). The highest prevalence of infertility was observed in 40-49 yr old group and the lowest in 20-29 yr old (Table III). 

Among infertile women, the infertility was 51.9% primary and 48.1% secondary. In 20-29 yr old group, out of the 31 women who were infertile, 64.5% had primary and 35.5% had secondary infertility, these ranges in the age group 30-39 yr were (out of 63 infertile), 46.03% primary infertility and 53.97% secondary infertility and ultimately in the age range of 49-40 yr out of 62 infertile, 52.4% had primary infertility and 47.6% had secondary infertility. Infertility had significant relationship with age (p=0.032), family history of infertility (p=0.001), education level (p=0.001), abortion history and/or still-birth (p=0.026), BMI (p=0.018) and waist circumferences (p=0.022). The relationship between infertility and smoking was not statistically significant (Table III). 

Also the infertility associated factors were tested by spearman correlation. In this study, infertility with age (r=0.051- p=0.001), educational level (r=-0.41, p=0.001), BMI (r=-0.012-p=0.47), waist circumference (r=0.027-p=0.062), history of abortion and/or still birth (r=0.029-p=0.001), and family history of infertility (r=0.121-p=0.001) were significantly correlated. Table IV shows the association of primary infertility with studied factors in this study which were not significant.

**Table I:** Baseline characteristics of studied sample

**Variable**		**Frequency (percentages)**
Age (yr)		
	29-20	700 (26.8)
	39-30	927 (35.5)
	49-40	984 (37.7)
Marital status		
	Married	2530 (96.9)
	Widow	54 (2.1)
	Divorced	27 (1)
Education		
	Primary school and less	395 (15.1)
	High school	821 (31.5)
	Diploma and graduate diploma	955 (36.6)
	BSc	394 (15)
	MSc and doctorate	46 (1.8)
BMI		
	Low weight <18.5	87 (3.3)
	Normal weight 24.9-18.5	811 (31.1)
	Over weight 29.9-24.9	1000 (38.3)
	Chubby 39.9-29.9	663 (25.4)
	Extreme obese >39.9	49 (1.9)
Waist circumference		
	Normal/under 88 cm	881 (33.8)
	Obese/over 88 cm	1729 (66.2)
Desire to have child		
	Yes	607 (23.3)
	I do not think so	340 (13)
	No	1506 (57.7)
Positive family history of infertility		
	Yes	484 (73.3)
	I do not know	214 (8.3)
	No	1912 (18.5)
Abortion and stillbirth history		
	Yes	603 (24.9)
	No	1818 (75.1)

BMI**: **Body mass index

**Table II:** Prevalence of infertility in studied sample

		**Frequency (percentages)**	**95% CI**
Infertility			
	Yes	135 (5.2)	4.3-6.1
	No	2217 (84.9)	83.4-86.2
Cause of infertility			
	Male	53 (2)	1.5-2.6
	Female	73 (2.8)	2.2-3.5
	Both	15 (0.6)	0.3-0.9
	Unexplained	44 (1.7)	1.2- 2.8
Infertility type			
	Primary	81 (2.6)	2.4-3.8
	Secondary	75 (2.1)	1.89-3.4

Data presented as n (%).

**Table III:** Related factors of infertility in studied sample

**Variable**		**Infertile**	**Fertile**	**p-value**
Level of education				
	Primary school and less	25 (18.5)	371 (15)	0.001
	High school	40(29.6)	758 (31)	
	Diploma and graduate diploma	48 (35.5)	907 (37)	
	BSc	19 (14.2)	374 (15)	
	MSc and doctorate	3 (2.2)	43 (2)	
Age (yr)				
	29-20	26 (19)	674 (27)	0.032
	39-30	49 (36)	877 (36)	
	49-40	60 (45)	924 (37)	
Body mass index				
	<18.5	5 (4)	68 (3)	0.018
	18.5-24.9	28 (21)	728 (31)	
	25-29.9	44 (32)	819 (35)	
	30-39.9	46 (34)	647 (27)	
	≥40	12 (9)	98 (4)	
Smoking				
	Yes	2 (1.4)	14 (0.7)	0.32
	Sometimes	1 (0.6)	23 (1)	
	Quitted	0 (0)	9 (0.3)	
	No	132 (98)	2429 (98)	
Waist circumference				
	Normal (<88 cm)	57 (42)	824 (33)	0.022
	Obese (>88 cm)	78 (58)	1651 (67)	
History of abortion and stillbirth				
	Yes	39 (33)	564 (24)	0.026
	No	79 (64)	1739 (76)	
Positive family history of infertility				
	Yes	62 (46)	423 (18)	0.001
	No	73 (54)	1914 (82)	

Data presented as n (%).

* Chi-square test

**Table IV:** Associated factors of primary and secondary infertility in studied sample

**Variable **		**Primary**	**Secondary**	**p-value**
Level of education				
	Primary school and less	16 (20)	10 (13)	0.673
	High school	21 (25)	23 (30)	
	Diploma and graduate diploma	25 (31)	28 (38)	
	BSc	16 (20)	11 (14)	
	MSc and doctorate	3 (4)	3 (5)	
Age (yr)				
	29-20	20 (25)	11 (14)	0.241
	39-30	29 (36)	34 (45)	
	49-40	32 (39)	30 (41)	
Body mass index				
	<18.5	2 (2.5)	1 (1.4)	0.05
	24.9-18.5	21 (26)	18 (24)	
	29.9-25	28 (35)	21 (28)	
	39.9-30	17 (20.5)	30 (40)	
	≥40	13 (16)	5 (6.6)	
Smoking				
	Yes	2 (2.5)	2 (2.6)	0.66
	No	79 (97.5)	73 (97.4)	
Waist circumference				
	Normal (<88 cm)	35 (43)	22 (29)	0.051
	Obese (>88 cm)	46 (57)	53 (71)	
History of abortion and stillbirth				
	Yes	22 (27)	28 (38)	0.125
	No	59 (73)	47 (62)	
Family history of infertility				
	Yes	37 (46)	27 (36)	0.072
	No	44 (54)	48 (64)	

Data presented as number (%).

Chi-square test

## Discussion

In this study, the prevalence of infertility and some related factors were studied. Based on the results of this study, the adjusted prevalence of total infertility in the population of women aged 20-49 yr old who lived in Yazd in 2013-2014 was 4.73% (3.94-5.59%). The prevalence of primary infertility was 2.68 (2.4-3.8%) and the prevalence of secondary infertility was 2.15 (1.8-3.4%). Of the 135 infertile women, 51.9% had primary infertility and 48.1% had secondary infertility. In the previous study conducted in Yazd province in 2009 by Aflatounian and colleagues, the prevalence of infertility was estimated about 5.52% (95%CI: 4.9-6.1%) which is slightly higher than our study (12). The sample size of the mentioned study was 5200 couples who were randomly selected from 10 rural and urban areas of Yazd province. Data collection from the sample of their study was conducted 2004-2005. Of the 170 infertile couples, the primary infertility rate was 3.48% and the secondary was 2.04% (12). The crude prevalence of infertility in our study was almost identical, but the standardized prevalence was slightly lower in our study.

The main difference between the two large epidemiological studies over a decade is that: The population studied in our study is only women between 20-49 yr old while Aflatoonian and colleagues considered 16-45 yr old women (12). We also questioned infertility with female cause or with both causes (male and female), while in the above study, the couples (both males and females), aged between 16 and 45 yr old were studied. A study conducted in Mazandaran province in Iran in 1999 reported the prevalence of 13.2% infertility among 2953 randomly selected couples (15). Considering the nature of the samples and the meaningful association of infertility with factors such as age and education this difference can be justified. Another study conducted on 380 married 20-49 yr old women in Gonabad (Khorasan Jonoobi province) in 2006, reported the prevalence of infertility 11.9%. The prevalence of primary and secondary infertility was 6.5% and 5.4%, respectively in that study (16). 

In a conducted study in 2010 by Hosseini and colleagues on 2,400 women aged 18-49 yr who were randomly selected in 4 provinces of Iran including Isfahan, Hormozgan, Golestan and Kermanshah, the prevalence of primary infertility was 2.3%, 6.8%, 3.4%, and 2.2% respectively. Also the Secondary infertility has been reported as 1.3%, 3.1%, 2.4% and 1.2% for provinces respectively (17). Finally, in an Iranian meta-analysis, which was conducted in 2015 and included 13 studies conducted in Iran during the period of 2003-2011, the prevalence of infertility among 55,658 people was reported 13.2% (95% CI: 8.0-18.3%). That primary infertility was 5.2% and secondary infertility was 3.2% (18). 

Comparing the estimated prevalence of past studies with our findings revealed lower prevalence of infertility in Yazd. Some probable reasons for this observation are lower age of marriage in Yazd, lower age range of this study and the sampling method of our study. According to a large study in China-2009, 7872 newly married couples followed up for 5 yr; the prevalence of infertility among the participants in the study was 5.2%. In this study, infertility refers to non-occurrence of pregnancy after 24 months of unprotected intercourse (19). The main difference between theirs and our research is the method and definition of infertility. In another study in Pakistan conducted on 2010, a total of 7628 women referring to the Department of Obstetrics and Gynecology at the Islamabad State Hospital for non-infertile reasons were studied. The prevalence of infertility was reported 7% (20). The entry of individuals to the above study was non-random, which differs from our research. In a study conducted in Turkey, the prevalence of infertility was elevated from 1.9% in 2008 to 4.1% in 2013 (21). 

In a population-based study in Scotland on 2009, 4466 women aged 31-50 yr were randomly selected. The study of Bushnik *et al* (22). Showed that the prevalence of primary infertility was 9.8% and secondary infertility was 7%, and factors associated with infertility, history of pelvic surgery, chlamydial infection, endometriosis, chemotherapy, long-term health problems and obesity with infertility.

In a study on all couples in Canada-2011 (examples based on the country's database and female partners), the prevalence of infertility was 11.5-15.7% (23). Considering the higher marriage age and the occurrence of abortion before marriage in areas such as Canada, the difference in the prevalence of their study with our research is predictable. A study conducted in Gambia-2017 on 2291 women aged 20-49 yr who referred to one of the women's clinics between 2006 and 2007, reported 328 infertile women (14.3%) (24). 

The prevalence was higher than our research, which can be due to factors such as; the gynecology clinic sampling, age and education, especially in geographic areas such as Africa, this difference in prevalence can be justified. Infertility had significant relationship with age, family history of infertility, education level, abortion history and still-birth, BMI and waist circumferences. None of the factors investigated with infertility type (primary or secondary) were significant. In a study conducted in Yazd province, there was a significant relationship between fertility decline and marriage age (12). In recent studies of infertility, the relationship between older age and fertility reduction was observed. In other words, age is a major determinant factor in the function of the reproductive system and the ovarian spontaneous cycles (25, 26). In our study, there was a significant relationship between infertility and body mass index and waist circumference. Obesity and overweight in women are rising and obesity has harmful effects on body systems, especially fertility, and recent studies showed that the prevalence of obesity in infertile women is high and women with obesity and overweight are at risk of developing fertility diseases (27). 

According to a study by Esmaeilzadeh and colleagues, women with infertility experience had a 4.8-fold increased risk of obesity and almost a 3.8-fold increased risk of being overweight compared to women without infertility (28). This connection was also justified in our research. In the present study, there was a significant relationship between infertility rate and education level. The prevalence rate of infertility was higher at the low and middle socio-economic level (12). This study has some limitations. First infertility was investigated by a questionnaire not specialist diagnosis which is a common limitation of most epidemiologic studies. Another item that can be mentioned is the age range of 20-49 yr for women who participated in the study. According to National Portal of Statistics of Iran in 2015, 25.2% of women under the age of 20 were married [14-19 yr old] (29). 

However, in the study of Vahidi and colleagues, in the Iranian population, it has been shown that the prevalence of infertility increases if the age of the first marriage is under 17 yr of age (30). The unavailability of the age of the first marriage in the current study can be one of its limitations. The main strength of this study was random sampling and large sample size. Also, the interviewers were well trained for asking relevant questions and measuring anthropometric variables with calibrated instruments.

## Conclusion

Our study reported that the standardized prevalence of infertility among women living in Yazd Greater Area is 4.73% which was lower compared to other regions of Iran and many other parts of the world. Also, the results of this study showed significant correlation between infertility and educational level, body mass index, waist circumference, history of abortion and stillbirth and family history of infertility. Since comprehensive studies in Yazd province were not performed to evaluate the role of each of the risk factors, it is suggested in the future, designing and conducting of each factors for analytical and experimental studies.
